# HDR Brachytherapy Dose Distribution is Influenced by the Metal Material of the Applicator

**DOI:** 10.1038/srep17863

**Published:** 2015-12-11

**Authors:** Chin-Hui Wu, Yi-Jen Liao, An-Cheng Shiau, Hsin-Yu Lin, Yen-Wan Hsueh Liu, Shih-Ming Hsu

**Affiliations:** 1 Institute of Nuclear Engineering and Science, National Tsing Hua University, Hsinchu, Taiwan, ROC; 2 School of Medical Laboratory Science and Biotechnology, Taipei Medical University, Taipei, Taiwan, ROC; 3 Department of Biomedical Imaging and Radiological Sciences, National Yang-Ming University, Taipei, Taiwan, ROC; 4 Medical Physics Research Center, Institute for Radiological Research, Chang Gung University. Chang Gung Memorial Hospital, Linkou, Taoyuan, Taiwan, ROC; 5 Department of Radiation Oncology, Saint Mary’s Hospital Luodong, Yilan, Taiwan, ROC; 6 Biophotonics and Molecular Imaging Research Center, National Yang-Ming University, Taipei, Taiwan, ROC

## Abstract

Applicators containing metal have been widely used in recent years when applying brachytherapy to patients with cervical cancer. However, the high dose rate (HDR) treatment-planning system (TPS) that is currently used in brachytherapy still assumes that the treatment environment constitutes a homogeneous water medium and does not include a dose correction for the metal material of the applicator. The primary purpose of this study was to evaluate the HDR ^192^Ir dose distribution in cervical cancer patients when performing brachytherapy using a metal-containing applicator. Thermoluminescent dosimeter (TLD) measurements and Monte Carlo N-Particle eXtended (MCNPX) code were used to explore the doses to the rectum and bladder when using a Henschke applicator containing metal during brachytherapy. When the applicator was assumed to be present, the absolute dose difference between the TLD measurement and MCNPX simulation values was within approximately 5%. A comparison of the MCNPX simulation and TPS calculation values revealed that the TPS overestimated the International Commission of Radiation Units and Measurement (ICRU) rectum and bladder reference doses by 57.78% and 49.59%, respectively. We therefore suggest that the TPS should be modified to account for the shielding effects of the applicator to ensure the accuracy of the delivered doses.

Statistics from the World Health Organization indicate that cervical cancer is the second most common cancer among women after breast cancer^1^. Therapies for cervical cancer include chemotherapy, external beam radiotherapy (EBRT) and brachytherapy. The continuing technological developments in medical instrumentation have resulted in radiotherapy providing patients with an improved cure rate and better post-treatment quality of life^2^,^3^.

Radiotherapy also damages the normal tissues surrounding a tumor. Thus, an important focus has been on increasing the tumor dose while reducing the adverse effects of the radiation on the surrounding normal tissue. According to the American Brachytherapy Society, a combination of EBRT followed by high dose rate (HDR) brachytherapy is currently the gold standard clinical practice for patients with cervical cancer treated using radiotherapy. A typical clinical treatment involves delivering an EBRT dose of 40 to 50 Gy to the entire pelvis over a 4- to 5-week period. HDR brachytherapy is then performed after EBRT with the dose applied in 3 to 10 fractions. The prescribed dose for each fraction is 4–8 Gy and is applied to point A of the Manchester system (i.e., 2 cm superior to the cervical opening)^4^. The organ reference dose defined by the International Commission of Radiation Units and Measurement Report 38 has been used to assess the probability of late sequelae^5^. Of patients who receive radiotherapy, 3–10% experience rectum and bladder sequelae such as proctitis, cystitis, and fistule^6^. The incidence rate increases with an increasing dose per fraction^7^,^8^.

Chen noted that the risk of bladder and rectum complications was high in patients whose ICRU bladder and rectum doses were greater than 24 and 16 Gy, respectively^9^. The authors suggested that the reference doses for both the bladder and rectum must be assessed to avoid late complications.

Brachytherapy for patients with cervical cancer involves positioning an applicator within the patient’s body, and the characteristics of the applicator material differ markedly from those of the soft tissues of the body. Henschke applicators have been widely used in patients with cervical cancer in recent years^10^,^11^. This type of applicator has an ovoid metal structure that is designed to reduce the doses applied to the bladder and rectum. However, the dose calculation formula in AAPM TG-43 (American Association of Physicists in Medicine Task Group No. 43) is based on the assumption of a homogeneous water environment^12^; the formula does not contain corrections for the inhomogeneous media inside the applicator or evaluate the dose distribution in water in the presence of the applicator. It is thus unclear whether the formula can be used to ensure the accuracy of the delivered doses.

The purpose of this study was to determine the effects of the shielding materials contained in the ovoid on the applied doses for multiple ^192^Ir source indwelling positions inside the Henschke applicator. This study used Monte Carlo N-Particle eXtended (MCNPX) code to simulate the dose distribution in water in the presence and absence of the Henschke applicator and made measurements using a thermoluminescent dosimeter (TLD). The values measured with the TLD were compared with the values calculated with the treatment planning system (TPS) and the MCNPX simulation results to explore the differences between the dose distributions in the presence and absence of the Henschke applicator as well as the doses at the ICRU bladder and rectum reference points.

## Results

### Comparison of the TPS calculation and MCNPX simulation values without considering the Henschke applicator

The 17 locations were selected for comparison. The MCNPX simulation and TPS calculation values at corresponding locations are listed in Table 1. The differences between these values were all less than 5%, with the exceptions of those at locations R2 and R4. The ratio of the TPS and MCNPX values ranged from 0.94 to 1.03. The results indicate that the two calculated values were in good agreement.

### Comparison of the TPS calculation, MCNPX simulation, and TLD measurement values when including the Henschke applicator

The dose in water in the presence of the Henschke applicator was simulated using MCNPX code, and the TLD measurement values were used to assess the dose distribution near the Henschke applicator. The dose differences among the TLD measurement, MCNPX simulation, and TPS calculation values are listed in Table 1. The greatest differences in doses between the MCNPX simulation and TLDs measured values were within 5%, with the ratio of the simulation and measured values ranging from 0.93 to 1.07. A comparison with the TPS calculation value indicated dose reductions from 89.33% to 1.89% for the TLD measurement value and from 76.49% to 7.16% for the MCNPX simulation value. Therefore, if the shielding effect of the Henschke applicator is neglected, the dose predicted by the TPS can be overestimated.

### 2D dose distribution and dose–volume histogram comparison

**Figure 1:**
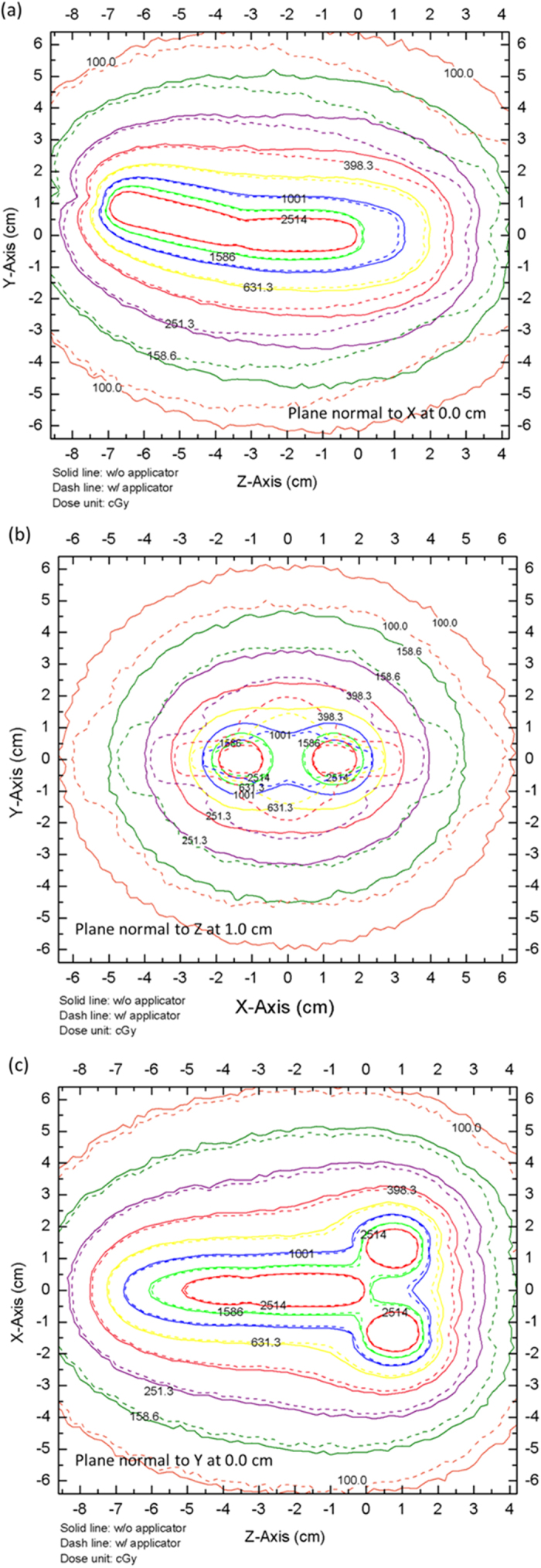
MCNPX calculated 2D dose distribution for the 17 source indwelling positions. Solid and dashed lines indicate dose distributions in the absence or presence of the Henschke applicator, respectively: (**a**) *z*-*y* plane at *x* = 0 cm, (**b**) *x*-*y* plane at *z* = 1.0 cm, and (**c**) *z*-*x* plane at *y* = 0 cm.

The 2D dose distribution calculated with the MCNPX code for the 17 source indwelling positions is shown in Fig. 1. The solid and dashed lines in the figure indicate the dose distributions in the absence and presence of the Henschke applicator, respectively. Figure 1(a) represents the *z*-*y* plane at *x* = 0 cm, which passes through the long axis of the tandem; Fig. 1(b) represents the *x*-*y* plane at *z* = 1.0 cm, which passes through the center of the ovoid; and Fig. 1(c) represents the *z*-*x* plane at *y* = 0 cm. The MCNPX results reveal that the 2D dose distribution was significantly reduced in the presence of the Henschke applicator, with the magnitude of the dose reduction being both location and distance dependent. The TPS calculation values do not account for the real dose distribution when the applicator contains metal shielding material.

**Figure 2:**
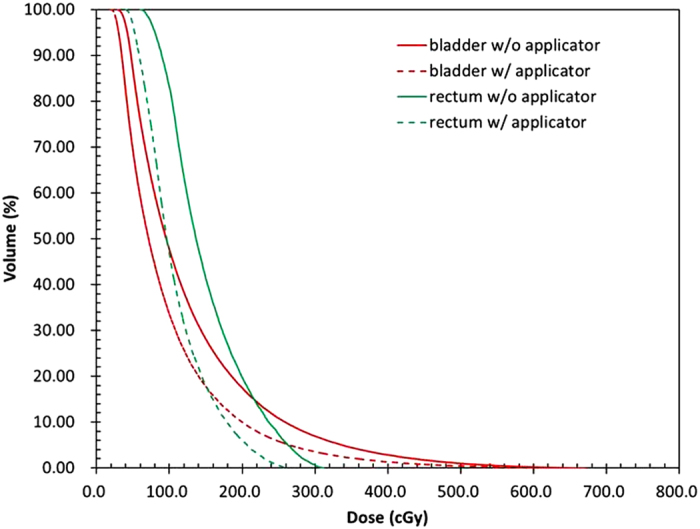
DVH comparisons for the rectum and bladder.

The dose differences in the bladder and rectum between the absence and presence of the Henschke applicator are listed in Table 2. When the Henschke applicator was present and the patient accepted single-fraction irradiation, the shielding material could reduce the bladder and rectum average doses by 30.32% and 38.95%, respectively. The dose–volume histograms (DVHs) of the rectum and bladder are shown in Fig. 2 with a spatial resolution of 1 mm^2^. Clearly, the presence of the Henschke applicator can reduce the rectum and bladder doses. The doses covering 50% and 20% of the organ volume (D_50_ and D_20_, respectively) decreased by 33.93% and 32.38% for the bladder between the presence and absence of the Henschke applicator, respectively, whereas they decreased by 41.90% and 37.98% for the rectum.

## Discussion

The TPS is used in brachytherapy when treating patients with cervical cancer. However, the TPS does not account for the presence of the Henschke applicator. To determine how the applicator affects the TPS calculation values, simulations were performed for an ^192^Ir source in the absence of the applicator for the 17 indwelling positions. The MCNPX and TLDs results were both consistent with the conclusion that the TPS would overestimate each point due to the presence of the Henschke applicator (Table 1). These results show that brachytherapy with a Henschke applicator may cause large dose differences when the TPS ignores the applicator.

The bladder (locations R8, R15, R16, and R17) and rectum (locations R9, R13, and R14) dose reference points were located above and beneath the Henschke applicator, respectively; these areas are in the shielding direction of the ovoid, and the doses in these areas were significantly reduced due to the shielding effect of the ovoid. The dose distribution and treatment quality will be affected if water is considered to be present at the location of the Henschke applicator. The metal material of the applicator used in brachytherapy should not be assumed to be the soft tissue. The results also imply that if the tumor is located behind the ovoid, the tumor dose will be overestimated if the shielding effect of the metal is not taken into consideration. A bladder accepting excessive radiation doses will produce incontinence, radiation cystitis, and hematuria, and the rectum will exhibit radiation proctitis, bloody stools, and chronic rectal ulcers^13^,^14^,^15^.

A typical clinical treatment plan was applied to the homemade water phantom to enable dose measurements and Monte Carlo simulations. The point dose measurements were consistent with the MCNPX simulation values. Our Monte Carlo simulation results accurately assessed the DVH and the critical organ doses, including at the ICRU rectum and bladder reference points (Tables 1 and 2 and Fig. 2). TG-43 recommends that the agreement between independent dose measurements and TPS dose calculations be within 15%. The MCNPX results indicated that the TPS overestimated the ICRU rectum and bladder reference doses by 57.78% and 49.59%, respectively. Thus, the TG-43 dose calculations produce unacceptable dose distributions when the Henschke applicator is present during brachytherapy.

The Henschke applicator has been widely used to treat patients with cervical cancer, and the present study assessed the effects of such an applicator on the dose distribution. When the TPS ignored the effect of the applicator, the MCNPX simulation values were consistent with the TPS calculation values. These results indicate that considering the Henschke applicator has relatively large effects on the dose distribution when brachytherapy is applied to patients with cervical cancer. The TPS currently appears to be insufficient in this case. This study thus recommends that the TPS be modified to take into account an applicator containing metal material to ensure the accuracy of the delivered doses.

## Methods

### ^192^Ir source and Henschke applicator

The ^192^Ir source used in this study had an active length of 3.6 mm, a diameter of 0.65 mm, and a density of 22.42 g/cm^3^; it was encapsulated by a stainless steel outer cover with an outer diameter of 0.9 mm that was welded to a steel cable for attachment to a remote after-loading machine (microSelectron-HDR v2, Nucletron, The Netherlands). The air kerma strength of this source is 45,730 cGy.cm^2^/h (11.2 Ci) when first installed, and the GENIE TPS (microSelectron, Nucletron) was used. In the Henschke tandem and ovoid applicator, the ovoid contains tungsten alloy as a shielding material with a density of 17.0 g/cm^3^ and the following atomic composition: 91% W, 4.5% Ni, and 4.5% Fe. The geometry of the shielding structure inside the ovoid was obtained with the aid of Kodak X-Omat V films.

### TLD and the dose readout system

To minimize the influence of the dose gradient on the measurements, the TLD-100 H cube (Harshaw, USA) with dimensions of 1 × 1 × 1 mm^3^ was used in this study. TLD-100 H (LiF: Mg, Cu, P) has an effective atomic number of 8.20 and a density of 2.64 g/cm^3^. The dose readings ranged between 1 μGy and 10 Gy in the TLD reading system (system UL-320, Rexon, USA).

### Homemade water phantom

**Figure 3:**
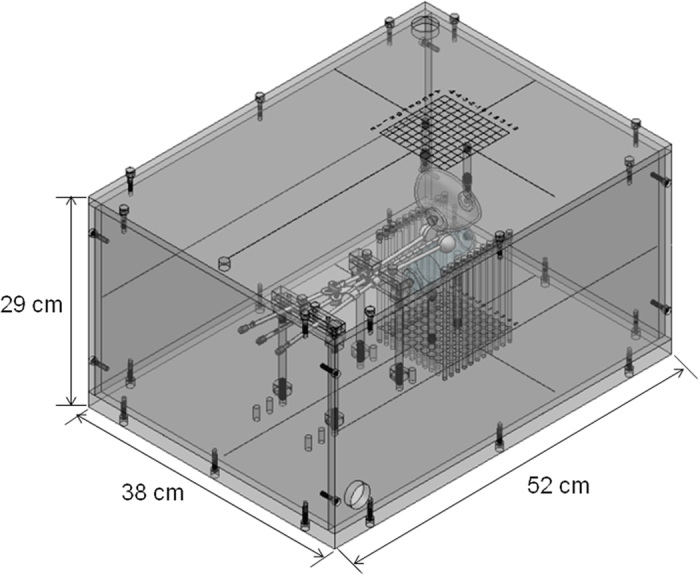
Photograph of the homemade water phantom (The figure was drawn by SM Hsu).

A water phantom hosted in an acrylic container was designed for brachytherapy dose measurements. The water phantom had dimensions of 52 × 38 × 29 cm^3^, 2-cm-thick top and button covers, and 1-cm-thick walls. Holes were drilled in the covers at 1-cm spacings, with 361 holes distributed in a matrix of 19 × 19 cm^2^, giving a total of 722 holes, as shown in Fig. 3. Acrylic rods of various lengths were constructed with a cavity to accommodate the TLD. The rods enabled the TLD to be placed in different holes for dose measurements. The rectum and bladder cavity phantoms were designed based on human anatomy, and the cavities were filled with water when performing the measurement experiments.

### Measurements with multiple source indwelling positions

Before performing the measurements, we referred to the Manchester system and ICRU38 report to determine the appropriate measurement points. The Manchester system of brachytherapy is the most widely used for cervical cancer and includes four points: A, B, rectum, and bladder. Point A is located 2 cm superior to the cervical opening and 2 cm lateral to the middle of the cervical canal, and point B is defined as being 3 cm lateral to point A. The ICRU report suggests that the reference points include those in the bladder, rectum, lymph trapezoid, and pelvic wall.

**Figure 4:**
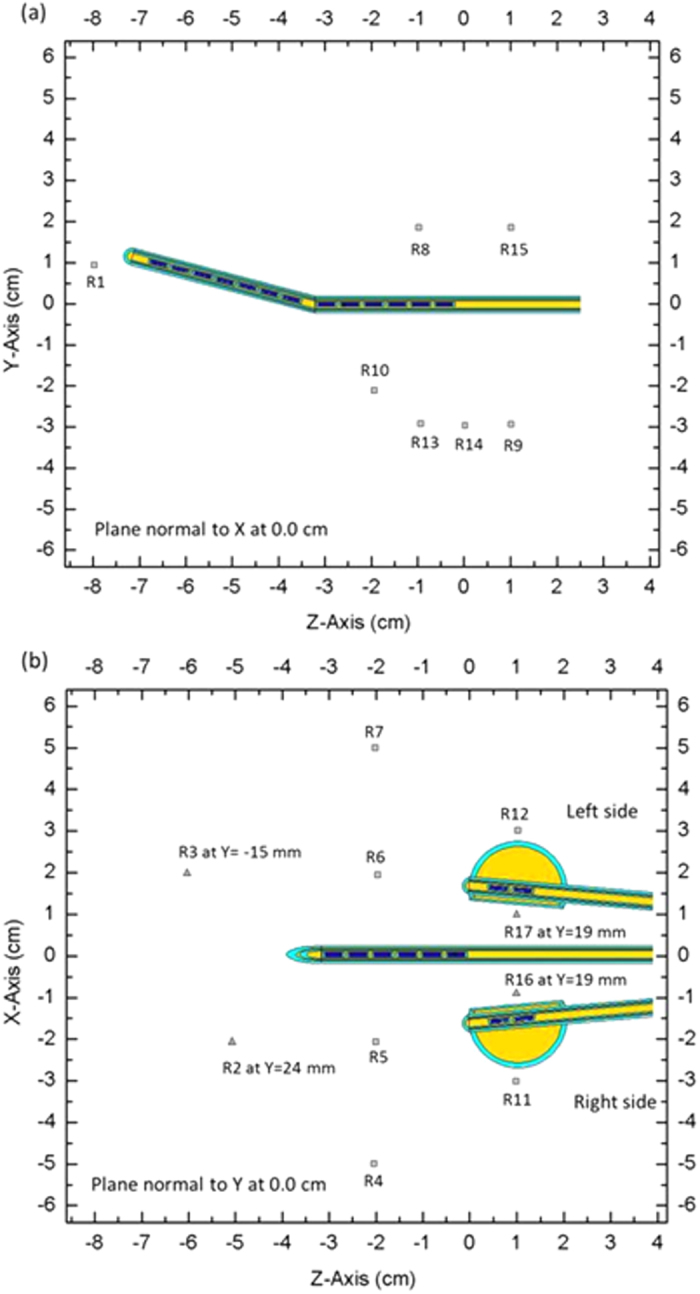
Dose measurement points when the Henschke applicator was present in the homemade water phantom. The squares indicate the measured points at *x* = 0 cm and *y* = 0 cm, and the triangles indicate the measured points in different planes: (**a**) *z*-*y* plane at *x* = 0 cm and (**b**) *z*-*x* plane at *y* = 0 cm. The *z* direction represents the foot-head direction, and the *y* direction represents the anterior-posterior direction.

In addition to the point A, point B, and ICRU bladder and rectum reference points, we added several measurement points in the region corresponding to the critical organ with the other points arranged along the tandem. This study used the orthogonal imaging method, which takes an anterior-posterior film and a lateral film to define the spatial coordinates of the source. This information enabled the TPS to be created, and a prescribed dose of 6 Gy was applied to point A for absolute dose comparisons. TLD readout values with variance coefficients less than 3% were selected to implement the dose measurement; the resulting 17 measurement points are indicated in Table 3 and Fig. 4.

### Monte Carlo simulation

The reliability of the TLD results was verified by comparisons with Monte Carlo simulations. The MCNPX code was developed by Los Alamos National Laboratory, and this study used MCNPX version 2.7.0 to calculate the ^192^Ir dose distribution^16^. The ^192^Ir photon spectrum was obtained from Brookhaven National Laboratory^17^. The simulation was divided into two parts: (A) simulations ignoring the Henschke applicator, with the simulation and TPS results being compared, and (B) simulations that included the Henschke applicator, which simulated the clinical treatment condition. The simulation results could be used to verify the TLD results and examine the accuracy of the TPS.

The mesh tally in MCNPX code was used to calculate the two-dimensional (2D) dose distribution around the ^192^Ir source. The mesh tally employed DE and DF cards to convert the fluence into the doses at particular points. The values of DE and DF are the energy bin and dose conversion factor, respectively, for a water medium. The mesh size for dose calculation was 1 × 1 × 1 mm^3^, with at least 10^8^ particles being simulated, which yielded 1σ statistical errors of less than 3% at all tested locations. Radiation particles were removed from the simulation when their energy was less than 10 keV.

The 17 source indwelling positions and indwelling times were simulated according to the TPS output file. To analyze the doses to the rectum and bladder, separate simulations were performed in the absence and presence of the Henschke applicator. A simple geometry was input to the MCNPX code to represent the rectum and bladder. The rectum comprised a hollow 7-cm-long and 0.2-mm-thick cylindrical shell with a diameter of 3 cm, and the bladder comprised a 0.2-cm-thick spherical shell with a diameter of 7.6 cm.

## Additional Information

**How to cite this article**: Wu, C.-H. *et al.* HDR Brachytherapy Dose Distribution is Influenced by the Metal Material of the Applicator. *Sci. Rep.* **5**, 17863; doi: 10.1038/srep17863 (2015).

## Figures and Tables

**Table 1:** Comparison of MCNPX simulation, TPS calculation, and TLD measurement values in the absence and presence of the Henschke applicator.

Position	w/o applicator			w/applicator				
	MCNPX (cGy)	TPS (cGy)	MCNPX/TPS	MCNPX (cGy)	TLD (cGy)	MCNPX/TLD	aDiff. between TPS & MCNPX (%)	bDiff. between TPS & TLD (%)
R1	377.70	374.92	1.01	313.66	308.78 ± 17.22	1.02	19.53	21.42
R2	320.97	340.47	0.94	294.43	277.56 ± 7.20	1.06	15.64	22.67
R3	282.48	296.64	0.95	276.61	289.61 ± 12.41	0.96	7.24	2.43
R4	162.48	173.73	0.94	146.84	150.79 ± 5.80	0.97	18.31	15.21
R5	576.49	598.68	0.96	510.74	502.52 ± 30.91	1.02	17.22	19.14
R6	574.14	599.43	0.96	536.07	546.08 ± 36.86	0.98	11.82	9.77
R7	162.56	170.69	0.95	159.29	150.67 ± 5.02	1.06	7.16	13.29
R8	616.68	641.50	0.96	428.85	402.76 ± 28.98	1.06	49.59	59.27
R9	289.60	290.84	1.00	184.33	178.87 ± 9.03	1.03	57.78	62.59
R10	531.72	547.12	0.97	478.33	495.80 ± 10.92	0.96	14.38	10.35
R11	525.09	547.88	0.96	483.87	486.35 ± 22.38	0.99	13.23	12.65
R12	489.01	488.49	1.00	453.76	479.41 ± 24.37	0.95	7.65	1.89
R13	337.55	352.84	0.96	297.50	308.59 ± 4.97	0.96	18.60	14.34
R14	324.11	332.39	0.98	236.38	253.77 ± 9.37	0.93	40.61	30.98
R15	502.99	487.78	1.03	332.45	319.39 ± 18.26	1.04	46.72	52.72
R16	501.50	490.04	1.02	277.65	258.83 ± 7.89	1.07	76.49	89.33
R17	489.85	477.27	1.03	279.45	278.58 ± 3.04	1.00	70.79	71.32

^a^(TPS-MCNPX)/MCNPX × 100%.

^b^(TPS-TLD)/TLD × 100%.

**Table 2:** Average dose, D_50_, and D_20_ in critical organs simulated using MCNPX code in the absence and presence of the Henschke applicator.

	Average Dose			D_50_			D_20_		
	w/	w/o		w/	w/o		w/	w/o	
Organ	(cGy)	(cGy)	Diff. (%)	(cGy)	(cGy)	Diff. (%)	(cGy)	(cGy)	Diff. (%)
bladder	99.17	129.24	30.32	71.45	95.69	33.93	140.17	185.56	32.38
rectum	108.51	150.78	38.95	96.32	136.68	41.90	144.35	199.17	37.98

**Table 3:** Measurement and calculated dose reference points in a realistic brachytherapy situation.

TLD	Measurement points	TLD	Measurement points
R1	Tandem 1	R10	Tandem 4
R2	Tandem 2	R11	Ovoid Lt
R3	Tandem 3	R12	Ovoid Rt
R4	Point B, Lt	R13	Rectum 1
R5	Point A, Lt	R14	Rectum 2
R6	Point A, Rt	R15	Bladder 1
R7	Point B, Rt	R16	Bladder 2
R8	ICRU Bladder point	R17	Bladder 3
R9	ICRU Rectum point		
